# Pro-environmental behavior in Iran using a systematic review and meta-analysis

**DOI:** 10.1016/j.heliyon.2021.e08424

**Published:** 2021-11-26

**Authors:** Jahangir Karami, Fateme Dehghan, Masoud Mohammadi

**Affiliations:** a Department of Psychology, Faculty of Social Sciences, Razi University, Kermanshah, Iran; b Cellular and Molecular Research Center, Gerash University of Medical Sciences, Gerash, Iran

**Keywords:** Environmental support behavior, Iran, Meta-analysis, Systematic review, Pro-environmental behavior

## Abstract

The problem of environmental pollution in today's world is not just a problem for the country or a specific territory. This research was conducted with the aim of investigating the environmental behavior of Iranian citizens. The present study was carried out using meta-analysis method on the performed studies (1998–2018) on pro-environmental behavior Papers related to the subject were gathered with the keywords for environmental behavior and environmental support behavior through searching on Magiran, SID, Scopus, Medline (PubMed) and Science-direct databases. Heterogeneity of studies was obtained using the I2 index, and data were analyzed in the comprehensive meta-analysis software. In 21 papers submitted to the meta-analysis process, 10 papers in the score range 20–100 (A) and 11 papers in the score range 1–5 (B), the total number of samples entered into the study was 3670 in the age range of 15–87 year scores (A), 4413 in the age range of 10–70 year scores (B). The overall mean and standard deviation of the environmental behavior (A) based on a meta-analysis of 50.4 ± 3.1 (44.56%–2.5%: 95% confidence limit) and in the score range 1–5 (B), 3.4 ± 0.18 (3.05%–5.7%, 95% confidence limit), were obtained. The effect of sample size and the publication issue of studies based on meta-regression in order to investigate the effect of heterogeneity in studies. The mean increases in each interval from 1 to 5, with an increase in sample size; in both intervals with an increase in the publication issue, the mean scores increase (P < 0.05). According to Mata-analysis results, the environmental behavior of Iranian citizens in both study intervals, is about average the global mean index of environmental performance.

## 1 Introduction

Today, the cause of many world environmental problems, such as the gradual warming of the earth, climate changes, air pollution, water scarcity, reducing the natural resources, and the destruction of natural ecosystem diversity, is rooted in human ecological behavior (Ghafourian and Hesari, 2017; Rashid and Mohammad, 2012). Environmental behavior is one of the new concepts that has been introduced today in the environmental studies of modern societies. The design of this concept in many environmental approach points to the importance of the role of these behaviors in the natural environment (Imamgholi, 2011). Conceptually, environmental behaviors are a set of community-based environmental actions that embraces a wide range of emotions, tendencies, and specific readiness for behavior toward the environment (Newton and Meyer, 2013). The environmental issues have been overlooked for many years; today, environmental issues have attracted the attention of the sociologists and psychologists due to the interdependence of the environment with the macro-human issues, including the culture, economic, development and politics, and much other material and spiritual aspects of human life (Van Wormer and Besthorn, 2017). In the last century, human-environmental behaviors are one of the most important and influential factors in the environment (Sookhtanlou and Vahedi, 2018). Environmental behaviors are affected by various factors, including cultural, economic, political, social, individual, and psychological factors. According to Stern's (2000) research, factors affecting the environmental behavior, including knowledge, skills, demographic variables, the behavior of general capabilities, habits of standard practice, and attitudes (norms, beliefs, and values), provide the public background for doing an environmental action that, in turn, it can affect all the environmental behaviors of the individual (Zsóka et al., 2013). The environmental literature review has identified the various factors impact the environmental behaviors such as the characteristics of respondents (Tarrant and Cordell, 1997; Zelezny et al., 2000), environmental awareness and concern, the concept of individual responsibility, the social norms of the narrative, positive attitudes (Olli et al., 2001; Bamberg and Möser, 2007), subjective norms, perceived behavior control, attitude, the tendency to behavior (Kalafatis et al., 1999; Mahon et al., 2006; Gadenne et al., 2011; Greaves et al., 2013; Botetzagias et al., 2015; Han, 2015), knowledge, attitudes and environmental values (Qi-yan and Yan-li, 2011; Molina et al., 2013), environmental attitude, place belonging and commitment to the environment and its preservation (Corral-Verdugo et al., 2003; Lee 2008; Imran et al., 2014), as the main psychosocial factors of environmental behaviors. Now, uncontrolled consumption of energy in Iran and subsequently the degradation of underground resources and environmental pollution can be among the most important issues related to people's environmental behaviors. Iran was the 13th largest producer of greenhouse gases in 2015, with a production of 4981.716 cubic meters (Olivier et al., 2015). The decline in forest cover over three decades from 21 million hectares to 14 million hectares and a comparison of the world's per capita share, Iran with 1 percent of the earth's surface area, accounted for only 0.36 percent of the world's renewable water resources; it is predicted to decline to 1,350 cubic meters per year in 2025. Also, by 2025, Iran will be placed under the boundary of the water crisis and the water stress border (Mohamad and Yazdanian, 2014). Iran has 16 million tons of soil erosion each year, six to eight million tons higher than the global mean average (Karimzadeh and Alizadeh, 2018), Iran with an area of about 165 million hectares in a dry climate, is limited to severe soil resources qualitatively and quantitatively (Sadeghi and Hazbavi, 2015).

The effects of population growth in the world have prompted farmers to excessively use agricultural land to produce the required food. Hence, human activities have been endangering and destroying the environment (Savari et al., 2021). Savari et al. (2021) introduced the determinants of the application of pro-environmental behaviors among Iranian farmers. The results revealed that about 59.8% of the variance of the farmers’ pro-environmental behavior were estimated using the technology acceptance model. Variables of attitude and intention, perceived ease of use, and perceived usefulness had significant effects on farmers’ pro-environmental behaviors. To explain pro-environmental behavior in the transport sector, Mehdizadeh et al. (2019) in their study showed that among the socio-demographic characteristics, parents in households who had more cars were less likely to choose sustainable transport modes. Also, accessibility to public transport had a positive effect on the choice of sustainable transport modes (Mehdizadeh et al., 2019). It was also shown in another study unfavorable attitudes toward safety and environment are positively associated with multimodal and monomodal car use among school children, latent factors play a mediating role between socioeconomic variables and modal groups. For instance, boys are negatively related to a weaker priority of safety in transport, which indirectly influences multimodality or monomodal, and unlike previous multimodality studies, the age of schoolchildren, car availability, and access to public transit are not found significant predictors of multimodal car use in school trips (Mehdizadeh and Ermagun, 2020).

In the 2018 ranking of the global environmental performance index, Iran ranked 80th among 180 countries (Wendling et al., 2018), and has been upgraded compared with 2016. This upgrade is promising and suggests that the environmental performance can be improved by some arrangements. Most studies have indicated that citizens' behavior in dealing with the environment around them, is an important factor in reducing the environmental degradations, and since the environmental behavior of Iranian citizens is unclear, the question of this study is how the environmental behavior of Iranian citizens. . In this regard, the present study was conducted with the aim of evaluating the environmental behavior of Iran's population through a systematic review and meta-analysis method based on the existing studies to determine its distance with the global mean average and the countries that have a favorable status in environmental behavior. The current study has been carried out in Iran in 2019.

## 2 Materials and methods

**Figure 1:**
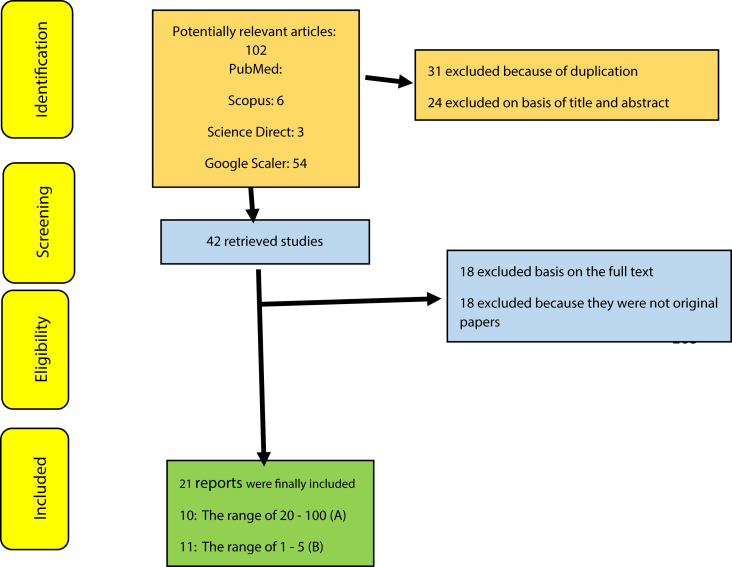
The flowchart on the stages including the studies in the systematic review and meta-analysis (Moher et al., 2009).

This study was conducted in a structured, systematic and meta-analytic review based on performed studies on the environmental behavior, including the papers published in Persian and English in national and international journals and searching on the Science direct, Medline (PubMed), Scopus, Magiran, and Scientific Information Database (SID) databases from March 1998 to December 2018. The search process in these databases was done using Persian keywords and their equivalent English words, including environmental behavior and pro-environmental behavior. Then all the papers related to the subject, which were conducted in different regions of Iran in Persian and English based on descriptive (cross-sectional) and interventional studies, and papers addressed to the other factors, were excluded from the list. The search studies were classified according to the environmental behavior scores in two scores range from 20 to 100 (A) and 1 to 5 (B) and were placed in the original list of papers. Then a checklist of the selected papers information, including the name of the researcher, the title of the article, the year of the study, the age range, the sample size, and the mean and standard deviation examined, and the quadruple stages of Preferred Reporting Items for Systematic Reviews and Meta-Analyses (PRISMA) Moher et al., and Prisma Group (2009) included the identification of the papers, the initial screening, eligibility, and, finally, the articles entered into the study (Figure 1). The review of the study was done using comprehensive meta-analysis software-version 3 (Neyeloff et al., 2012), and the information about these studies was compiled according to the researcher's affiliations, article title, year of study, age range, sample number, mean and standard deviation in Table 1.

**Table 1:** Pro-environmental behavior studies in Iran.

	Row	Participants' Age	Number of participants in each study	Mean ± SD	References
A: (The range of 20–100)	1	-	383	46.77	Salehi and Karemzadeh (2014)
	2	>18	400	68.22 ± 10.34	Mirfardi et al. (2017)
	3	18–70	400	46.84 ± 11.18	Novah et al. (2011)
	4	15–66	400	41.18 ± 9.68	Mirfardi, 2016
	5	-	384	40.65 ± 8.70	Vahida et al. (2017)
	6	18–60	384	60.81	Haghighatian et al., 2016
	7	-	375	27.82 ± 6.01	Shobeyri (2017)
	8	-	384	64.21 ± 8.03	Zareshahabadi et al. (2016)
	9	-	250	55.77 ± 14.06	Yazdani and Shams (2016)
	10	20–87	310	52.04 ± 7.15	Hejazi and Eshaghi (2014)
B: (The range of 1–5)	1	16	350	3.82 ± 0.55	Salehi and Ghaemi Asl (2013)
	2	>15	410	3.1 ± 0.88	Ahmadian Haghighatian (2016)
	3	>15	410	3.1 ± 0.88	Haghighatian et al. (2015)
	4	>10	376	3.08 ± 0.47	Haghighatian (2014)
	5	15–70	400	3.38 ± 0.42	Mokhtari et al. (2014)
	6	17	375	3.72 ± 3.54	Mazloumian et al. (2016)
	7	>18	400	2.66	Ghaderi et al. (2015)
	8	>18	440	3.90 ± 0.52	Salehi and Imamgholi (2012)
	9	19–52	400	3.52	Hemayatkhah Jahromi, Ershad, Danesh, Ghorbani (2017)
	10	18–48	385	3.47 ± 0.55	Salehi and Imamgholi (2012)
	11	>18	467	3.85	Salehi and Imamgholi (2016)

### 2.1 Environmental behavior

Conceptually, the environmental behavior refers to the clear and visible actions that are performed by the individual and in response to the environment (He et al., 2011). Operationally, the environmental behaviors are evaluated in 20 items with a 5-point scale, the coding method is as follows: “very high, 5”, “relatively high, 4”, “average score, 3”, “relatively low, 2”, and “very low, 1”, Considering that in the studied studies, this criterion has been considered by the studies and this criterion has been used the most, so the authors have also considered this criterion. Considering that in the previous studies, this criterion has been considered by scientists and this criterion has mostly been used, so the authors have also considered this criterion. Article review and data extraction activities were performed by two reviewers (JK, FD), independently. If an article was not included, the reason for excluding it was mentioned. In cases where there was a disagreement between the two reviewers, a third person (MM) reviewed the article (He et al., 2011). Cronbach's alpha of the items of environmental behavior has been measured at 0.77 (Mirfardi et al., 2017). Iran is the 18th largest country in the world with an area of 1648,195 square kilometers. Iran is located in the northeast hemisphere of the Asia continent and in the western part of Iran plateau and is one of the Middle East countries. It is bordered by Armenia, Azerbaijan, and Turkmenistan in the north, Afghanistan, and Pakistan in the east, and Iraq and Turkey in the west. In addition, Iran has a blue border with Kuwait, Iraq, Saudi Arabia, Bahrain, Oman, Qatar, and the United Arab Emirates in the Gulf. Despite the large area, only 14% of Iran's lands is cultivated, 8% is forest, 55% is natural grasslands and 23% are deserted. It has a variety of climatic conditions so that among the 13 known climates in the world, 11 are in Iran (Yousefi et al., 2010).

**Figure 2:**
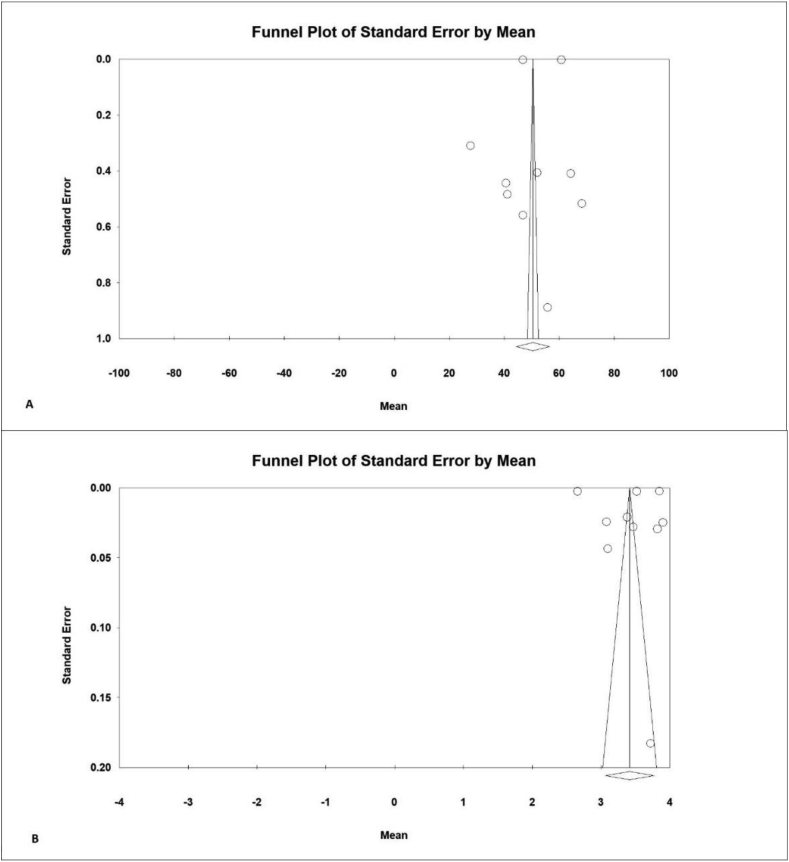
Funnel Plot of the mean of environmental behavior scores, 20 to 100 (A) and 1–5 (B).

In each study, the rate of environmental behavior was obtained; the heterogeneity of the studies was evaluated using I^2^ ​test. In general, heterogeneity is classified into three categories, the heterogeneity less than 25% (low heterogeneity), between 25% and 75% (medium ​heterogeneity) and above 75% (high heterogeneity), which, according ​to the results in the range of 20–100 (I^2^ = 99%) (A), and in the range of 1–5 (I^2^ = 99%) (B), and high heterogeneity in the studies, the random effects model was used to combine the results of the studies. Data were analyzed using Comprehensive Meta-analysis software (Neyeloff ​et al., ​2012). The probability of publication bias of the results was estimated by the funnel chart (Figure 2), using the Egger test and the significance level of 0.5 for both ranges of environmental behavior scores, 20 to 100 (A) and 1–5 (B). It indicates that the publication bias was not statistically significant (P = 0.979) (A) and (P = 0.998) (B).

## 3 Results

**Figure 3:**
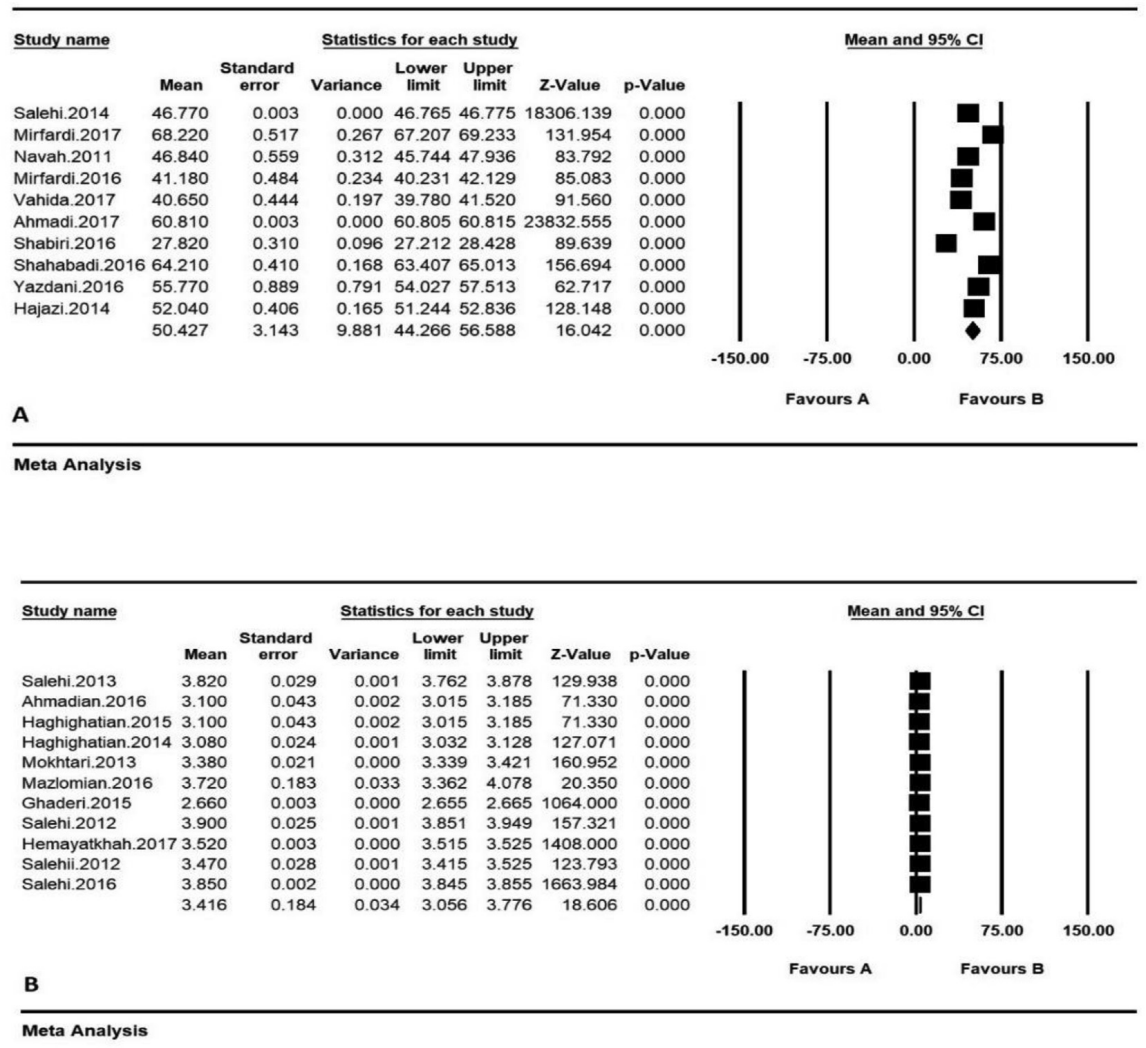
Total mean and standard deviation of environmental behavior scores, 20 to 100 (A) and 1–5 (B).

**Figure 4:**
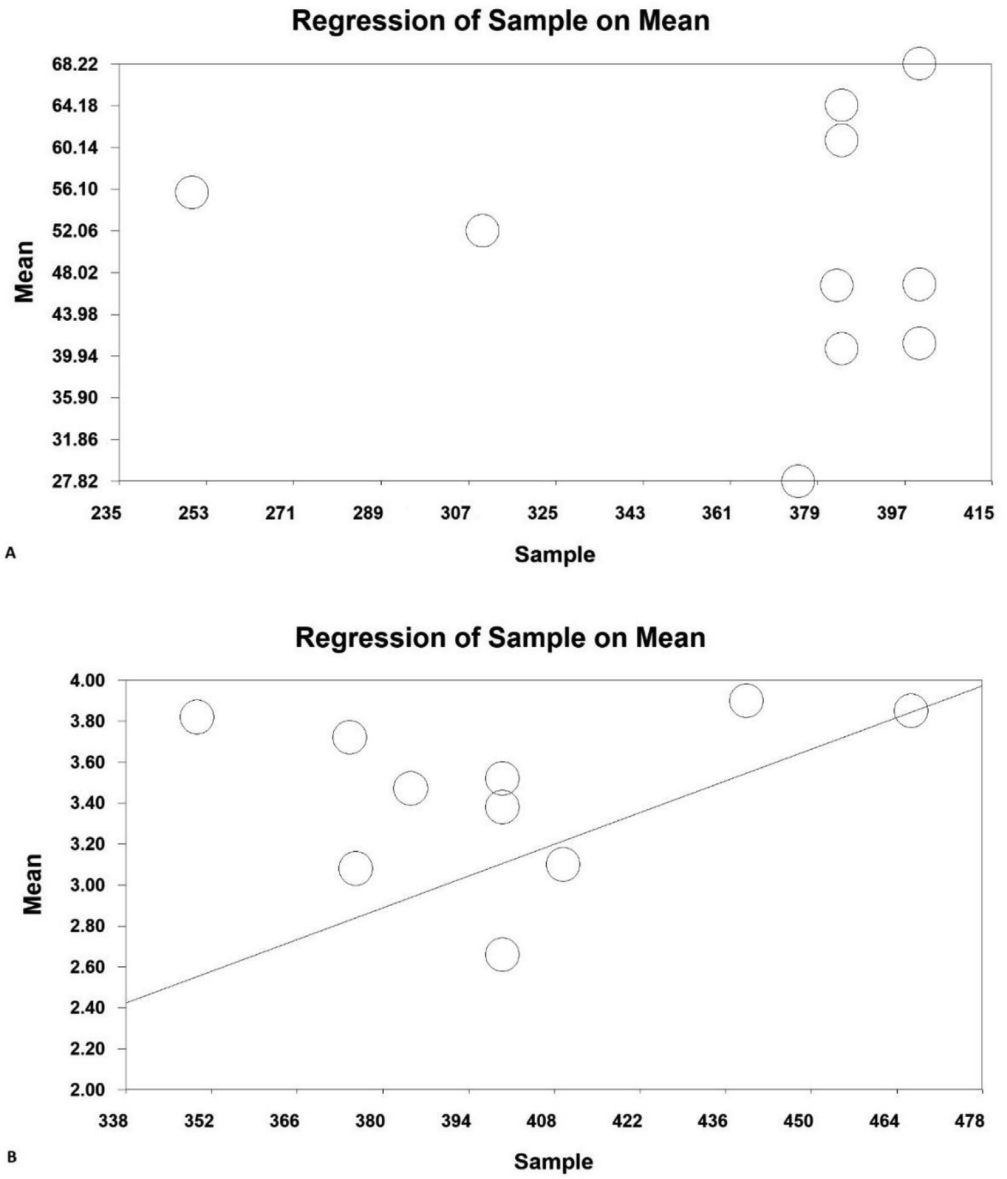
The total results of environmental behavior scores, 20 to 100 (A) and 1–5 (B).

**Figure 5:**
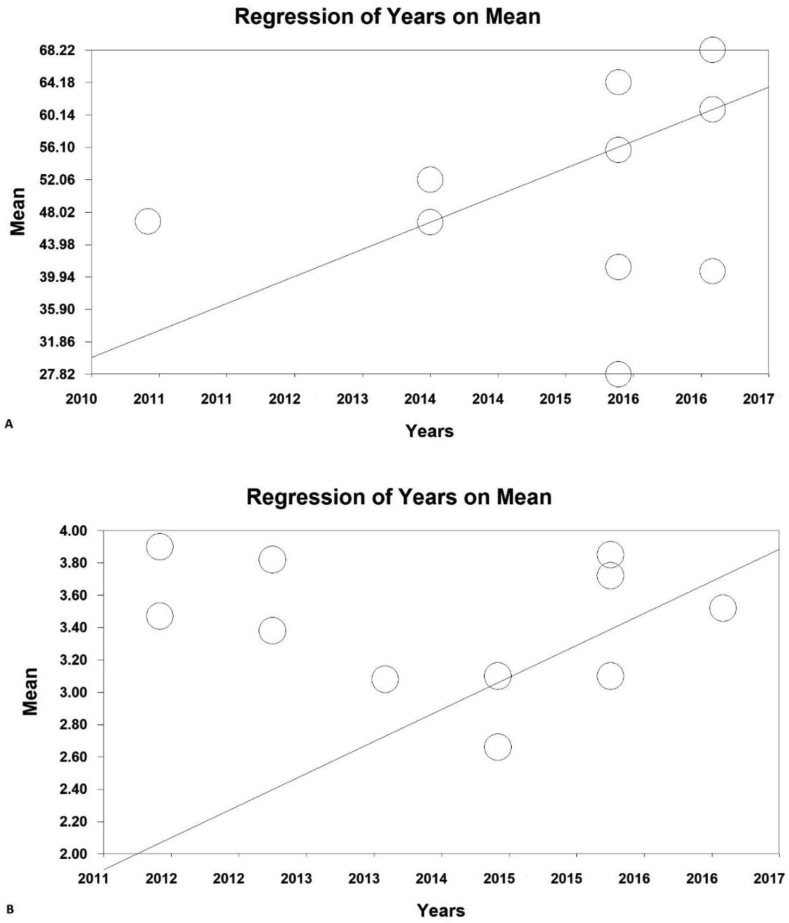
Total results of environmental behavior scores, 20 to 100 (A) and 1–5 (B).

According to the researches done on Iranian's average environmental behaviors, including the papers published in national and international journals, 38 papers were obtained through searching the Scientific Information Database (SID) and Magiran, 3 studies in Science Direct, 6 studies in Scopus, and 50 studies from Google Scholar search engines. Then among the papers with initial condition for entry into the study, 42 studies were selected based on preliminary studies, and with the elimination of 55 repetitive papers. Eventually, with eliminating 18 unrelated papers and removing 14 studies over the secondary studies due to the lack of abstract and the main paper, as well as the poor quality of the papers, 21 papers were obtained. According to the classification of the range of environmental scores, 10 studies in the scores range from 20 to 100 (A) and 11 studies in the range of environmental scores from 1 to 5, were separately introduced into the meta-analysis process (Figure 1). The total number of samples entered into the study was 3670 in the range of scores (A). The overall mean of environmental behavior in the range of scores 20 to 100 (A) was obtained based on meta-analysis (50.4 ± 1.3) (44.2–5.54%: 95% confidence interval); the highest mean was obtained in Shahabadi and Feyzi (2016) study 0.41 ± 64.2 (63.4–65%,95% confidence interval) and the lowest mean in Shobeyri's (2017) study was 27.8 ± 0.31 (27.2–28.4%, 95% confidence interval). The overall mean of environmental behavior (B) in the range of scores 1–5 was obtained based on meta-analysis of 3.4 ± 0.18 (3.5–3.7% confidence interval: 95% confidence interval); the highest mean in Salehi and Imamgholi (2012) study was obtained 3.9 ± 0.02 (3.8 ± 3.94%: 95% confidence interval) and the lowest mean in Ghaderi et al. (2015) study was obtained 2.66 ± 0.003 (2.66–2.65%), the 95% confidence interval (Figure 3). In Figure 3, the mean of the random effects model is presented, in which the black square is the mean and the square length is 95% confidence interval in each study, the rhizome sign represents the total mean in all studies. In order to investigate the effects of the potentially effective factors on the heterogeneity of the mean, the Meta regression was used for two sample size and the publication issue (Figure 5). According to Figure 4, with the increase of sample size, the overall mean increases from 1 to 5 in the range of environmental behavior scores which has a statistically significant difference (B) (P < 0.05); however, in the range of environmental behavioral scores 20–100 incremental and decreasing effects were not observed (A). in Figure 5, it was also reported that in both range of environmental behavior scores of 20–100 (A) and the overall mean of environmental behavior scores of the range 1–5 (B), increases with the increase in the research year, and this difference was statistically significant (P < 0.05).

## 4 Discussion

One of the most important issues of the twenty-first century is the environmental issues. Solving Iran's environmental problems requires planning and providing solutions. The aim of this study was to evaluate the environmental behavior in Iran that based on the meta-analysis of the existing studies, the average overall of environmental behavior was obtained at the global mean average level. In a study done by Fang et al. (2018), the mean environmental behavior of Taiwanese farmers younger than 40 years old was 3.68 ± 0.88, and for the farmers older than 40 years old, it was 56.3 ± 0.84.

Also in China, the environmental behavior of citizens was reported 0.74 ± 3.50 (Li et al., 2019). In Canada, this level was 4.010 ± 0.92 for Consumer PEBs and 1.61 ± 70 for Activist PEBs (Schmitt et al., 2019). According to the report of 2018, the Global Environmental Performance Index (EPI), Switzerland, France, Denmark, Malta, Sweden, the UK, Luxembourg, Australia, Ireland and Finland are 10 countries that have had the best environmental performance. Iran ranked mid-level with 80th among 180 countries, thus more strategies are needed to improve the environmental performance index. Accountability makes the person sensitive to the environment and its problems and works to solve the environmental problems. In fact, it can be argued that the level of the individual's adherence to environmental behaviors becomes higher with increasing the accountability. Bourdieu (1999) analyzed the type of person's behavior, according to the individual's class position and the type of economic and cultural capital. The behavioral tastes analyzed by Bourdieu (1999) can largely explain the practical and objective conditions of human behavior with the surrounding environment. Each person comes to a certain type of social attitude and action depending on the class position, his assets, and the area of interacting with the light of his class position in society (Moghimehfar and Halpenny, 2016).

Obviously, many factors and variables are involved in the process of shaping behavior; various factors such as situational factors, cultural and social context, socialist methods, knowledge, attitude and approach of individuals to the topic, all affect the formation of particular behavior. For this reason, moving towards a greater understanding of the complexities of values, attitudes, areas, and personality factors that affect the particular behavior, is necessary (Davis et al., 2015). Situational variables, psychological variables and environmental values together, are three factors influencing the environmental behavior. One of the most important problems of the today's world is the destruction of the environment and its effects on people's lives. It is believed that, humans have to behave more responsibly towards the environment in order to minimize these problems and threats. Responsible environmental behaviors are a set of actions of community members towards the environment. The sense of social responsibility is an important variable in society for the proper organization of all social behaviors, and especially for the environment. Individuals who behave responsibly and indifferently in society, do not focus solely on the short-term period and are not solely concerned with their individual interests, but behave proportional to the assessment of individual and social consequences, as well as with the respect of the general interests of the society.

Finally, it can be said that reducing individuality and increasing the participation of individuals and strengthening their sense of social responsibility, makes people feel belonging to the environment and behave more appropriately to the environment.

## 5 Conclusion

According to the results of this study, Iranian people are at the average of the global mean average in terms of environmental behaviors in both areas of the examined scores. Since the environmental issues have become one of the most important issues and challenges of the 21st century, and reducing its consequences requires the serious determination, cooperation, mutual understanding and awareness of it, applying some strategies to increase citizens' environmental behavior and consequently reducing the environmental impacts, can be very helpful.

The constraints of the present research were the lack of access to the full text of some papers and the poor quality of some papers under review. Thus, further results based on this methodology should be conducted to corroborate the present results in these and in other countries all over the world.

## Declarations

### Author contribution statement

Jahangir Karami: Conceived and designed the study; Analyzed and interpreted the data; Contributed reagents, materials, analysis tools or data; Wrote the paper.

Fateme Dehghan: Performed the experiments; Analyzed and interpreted the data; Wrote the paper.

Masoud Mohammadi: Contributed reagents, materials, analysis tools or data; Wrote the paper.

### Funding statement

This work was supported by 10.13039/501100006766Iran University of Science and Technology (97013613).

### Data availability statement

Data included in article/supplementary material/referenced in article.

### Declaration of interests statement

The authors declare no conflict of interest.

### Additional information

No additional information is available for this paper.
